# Supplementary material for: A Metabolomic Study of the Variability of the Chemical Composition of Commonly Consumed Coffee Brews
Source: Metabolites. 2019 Jan 18;9(1):17. doi: 10.3390/metabo9010017 (PMC6359601; doi:10.3390/metabo9010017)
Supplement: Supplementary file 1 [file metabolites-09-00017-s001.zip › final-suppl/metabolites-404599-revised-suppl.pdf]

Online supplementary information

A metabolomic study of the variability of chemical composition of commonly consumed coffee brews

Joseph A Rothwell[1, §], Erikka Loftfield[2, §], Neal Freedman[2], Callie Kambanis[2], Augustin Scalbert[1,*], Rashmi Sinha[2]

[1]International Agency for Research on Cancer (IARC), Nutrition and Metabolism Section, Biomarkers Group, 150 Cours Albert Thomas, F-69372 Lyon Cedex 08, France

[2]National Cancer Institute, 9609 Medical Center Drive, Bethesda MD 20892, USA

Online Supplementary Information

Table of Content

Tables

Table S1: Summary of mass spectral data supporting peak annotations in coffee brews.

Table S2. Brewed or purchased coffee samples and their characteristics.

Table S3. Coefficients of variation for the coffee compounds identified or tentatively identified.

Figures

Figure S1. PC-PR2 analysis of of metabolomic data from the coffee brew samples, Principal components explaining >80% of the variability were selected.

Figure S2. Boxplots showing means of sums of total intensities of mass spectrometry signals (n = 3670) for each type of coffee brews.

Figure S3. Principal component analysis of constituent profiles of the 18 annotated coffee compounds measured in 76 coffee brew samples. Scores on PC2 and PC3 are shown. (A) Color-coded according to type of coffee brew. (B) Color-coded according to decaffeination. (C) Loading plots.

Figure S4. T-tests comparing different groups of coffee brew samples. Dark line indicates the limit for significant difference after Bonferroni correction. Comparison of dark roasted brews with medium roasted brews, decaffeinated and caffeinated coffee brew samples, coffee brews prepared with Arabica beans and blends of Arabica and Robusta beans; instant coffees to all other coffee brew samples.

Figure S5. Heatmap showing correlations between annotated coffee compounds.

Supplementary spreadsheet (see separate Excel File)

Supplementary Spreadsheet: Chromatograms and spectra supporting annotation of compounds.

Table S1: Summary of mass spectral data supporting peak annotations in coffee brews.

Assigned identity 1	Formula	Observed m/z	Ion	Mass difference (delta ppm)	Retention time (min)	Annotation l evel[1]
1-(2-Furanyl)-1,2-butanedione *	C8 H8 O3	153.0552	[M+H]+	0.37	3.11	4
Unknown		335.2223	[M+H]+		5.64	5
Unknown		335.2223	[M+H]+		5.49	5
3,4-Dicaffeoylquinic acid lactone *	C25 H22 O11	499.1241	[M+H]+	15.03	3.2	4
Unknown		110.0607	[M+H]+		2.51	5
Unknown		110.0607	[M+H]+		3.42	5
Unknown		101.0603	[M+H]+		1.97	5
Unknown		125.0716	[M+H]+		2.23	5
Unknown		125.0716	[M+H]+		2.32	5
Unknown		280.1550	[M+H]+		2.59	5
Unknown		103.0396	[M+H]+		2.12	5
3,4-Dimethyl-1,2-cyclopentanedione *	C7 H10 O2	127.0760	[M+H]+	8.09	3.03	4
3,5-Dicaffeoylquinic acid	C25 H24 O12	517.1347	[M+H]+	0.63	3.85	1
3-Caffeoylquinic acid	C16 H18 O9	355.1030	[M+H]+	0.62	2.94	1
5-Caffeoylquinic acid	C16 H18 O9	355.1030	[M+H]+	0.90	2.46	1
5-Hydroxymethylfurfural *	C6 H6 O3	127.0396	[M+H]+	6.30	2.09	4
alpha-Furyl methyl diketone isomer (i) *	C7 H6 O3	139.0396	[M+H]+	15.90	1.86	4
alpha-Furyl methyl diketone isomer (ii) *	C7 H6 O3	139.0396	[M+H]+	15.90	2.13	4
Atractyligenin *	C19 H28 O4	321.2067	[M+H]+	0.75	5.37	4
Cafamarine	C26 H36 O10	509.2387	[M+H]+	0.50	4.05	3
Unknown		371.0979	[M+H]+		2.94	5
Unknown		371.0979	[M+H]+		2.46	5
Unknown		351.1081	[M+H]+		3.83	5
Unknown		165.0552	[M+H]+		3.02	5
Unknown		125.0603	[M+H]+		3.35	5
Cafestol *	C20 H28 O3	317.2117	[M+H]+	2.39	5.49	4
Caffeoylquinic acid isomer (i) *	C16 H18 O9	355.1030	[M+H]+	0.62	3.01	4
Unknown		87.0447	[M+H]+		2.09	5
Caffeoylquinic acid isomer (ii) *	C16 H18 O9	355.1030	[M+H]+	1.46	2.8	4
Caffeoylquinic acid isomer (iii)*	C16 H18 O9	355.1030	[M+H]+	0.90	2.21	4
Unknown		181.0502	[M+H]+		2.42	5
Caffeoylquinic acid lactone isomer (i)*	C16 H16 O8	337.0924	[M+H]+	2.03	3.5	4
Caffeoylquinic acid lactone isomer (ii)*	C16 H16 O8	337.0924	[M+H]+	1.44	3.36	4
Cinnamoylglycine *	C11 H11 N O3	206.0818	[M+H]+	5.40	4.09	4
Coixinden A *	C11 H10 O3	191.0709	[M+H]+	2.66	4.45	4
Coumaroylquinic acid isomer (i)*	C16 H18 O8	339.1081	[M+H]+	1.97	3.35	3
Coumaroylquinic acid isomer (ii)*	C16 H18 O8	339.1081	[M+H]+	3.65	2.82	4
Coumaroylquinic acid lactone *	C16 H16 O7	321.0975	[M+H]+	4.36	3.72	4
Cyclo(isoleucyl-prolyl)	C11 H18 N2 O2	211.1447	[M+H]+	3.67	3.76	2
Cyclo(isophenylalanyl-prolyl)	C14 H16 N2 O2	245.1291	[M+H]+	5.01	3.94	2
Cyclo(leucyl-phenylalanyl) *	C15 H20 N2 O2	261.1604	[M+H]+	3.16	4.55	4
Cyclo(leucyl-prolyl)	C11 H18 N2 O2	211.1447	[M+H]+	3.67	3.87	1

Cyclo(phenylalanyl-prolyl)	C14 H16 N2 O2	245.1291	[M+H]+	3.78	4.05	1
Cyclo(prolyl-prolyl) *	C10 H14 N2 O2	195.1134	[M+H]+	5.52	2.6	4
Cyclo(prolyl-valyl)	C10 H16 N2 O2	197.1291	[M+H]+	3.17	3.08	1
Unknown		85.0654	[M+H]+		1.89	5
Unknown		234.1495	[M+H]+		2.59	5
Unknown		87.0447	[M+H]+		1.48	5
Dicaffeoylquinic acid isomer *	C25 H24 O12	517.1347	[M+H]+	1.21	4.12	4
Unknown		195.0658	[M+H]+		3.8	5
Feruloylquinic acid isomer (i)	C17 H20 O9	369.1186	[M+H]+	1.27	3.47	3
Feruloylquinic acid isomer (ii)	C17 H20 O9	369.1186	[M+H]+	1.27	2.99	3
Unknown		99.0447	[M+H]+		1.86	5
Guanosine*	C10 H13 N5 O5	284.0996	[M+H]+	1.52	1.66	4
Methyl-2-pyrrolecarboxaldehyde	C6 H7 N O	110.0607	[M+H]+	4.00	3.62	1
N-caffeoyltryptophan	C20 H18 N2 O5	367.1295	[M+H]+	1.99	4.48	2
N-p-Coumaroyltryptophan	C20 H18 N2 O4	351.1346	[M+H]+	2.41	4.71	2
Unknown		149.0451	[M+H]+		1.42	5
Paraxanthine	C7 H8 N4 O2	181.0726	[M+H]+	10.41	2.69	1
Sotolone *	C6 H8 O3	129.0552	[M+H]+	1.22	2.72	4
Theobromine	C7 H8 N4 O2	181.0726	[M+H]+	3.75	2.37	1
Unknown		193.0502	[M+H]+		3.74	5
Theophylline	C7 H8 N4 O2	181.0726	[M+H]+	4.30	2.81	1
Unknown		127.0396	[M+H]+		2.6	5

[1]Confidence level for identification: see Materials and Methods.
* Hypothetical annotations.

Table S2. Brewed or purchased coffee samples and their characteristics.

Coffee brand and brew method[1]	Caffeine	Bean type	Roast	Place of purchase or preparation
**Brand 1 (n = 2)**				
Boiled	Caffeinated	Arabica	Dark	Home
**Brand 2 (n = 4)**				
Cold Brew	Caffeinated	Blend	Medium	Laboratory
Filter	Caffeinated	Blend	Medium	Laboratory
French Press	Caffeinated	Blend	Medium	Laboratory
Percolated	Caffeinated	Blend	Medium	Laboratory
**Café or restaurant 1 (n = 9)**				
Cold Brew	Caffeinated	Arabica	Medium	Laboratory
Espresso	Caffeinated	Arabica	Dark	Restaurant
Espresso	Decaffeinated	Arabica	Dark	Restaurant
Filter	Caffeinated	Arabica	Medium	Restaurant
Filter	Caffeinated	Arabica	Medium	Laboratory
Filter	Decaffeinated	Arabica	Medium	Laboratory
Filter	Decaffeinated	Arabica	Medium	Restaurant
French Press	Caffeinated	Arabica	Medium	Laboratory
Percolated	Caffeinated	Arabica	Medium	Laboratory
**Brand 3 (n = 1)**				
Filter	Caffeinated	Arabica	Dark	Laboratory
**Brand 4 (n = 12)**				
Cold Brew	Caffeinated	Blend	Medium	Laboratory
Filter	Caffeinated	Blend	Dark	Laboratory
Filter	Caffeinated	Blend	Light	Laboratory
Filter	Caffeinated	Blend	Medium	Laboratory
Filter	Decaffeinated	Blend	Medium	Laboratory
French Press	Caffeinated	Blend	Medium	Laboratory
Instant	Caffeinated	Blend	Medium	Laboratory
Instant	Decaffeinated	Blend	Medium	Laboratory
K-Cup	Caffeinated	Arabica	Medium	Laboratory
K-Cup	Decaffeinated	Arabica	Medium	Laboratory
Percolated	Caffeinated	Blend	Medium	Laboratory
Filter	Caffeinated	Blend	Dark	Laboratory
**Brand 5 (n = 8)**				
Cold Brew	Caffeinated	Arabica	Medium	Laboratory
Filter	Caffeinated	Arabica	Dark	Laboratory
Filter	Caffeinated	Arabica	Light	Laboratory
Filter	Caffeinated	Arabica	Medium	Laboratory
Filter	Decaffeinated	Arabica	Medium	Laboratory

French Press	Caffeinated	Arabica	Medium	Laboratory
Percolated	Caffeinated	Arabica	Medium	Laboratory
Filter	Decaffeinated	Arabica	Medium	Laboratory
**Brand 6 (n = 1)**				
K-Cup	Caffeinated	Arabica	Medium	Laboratory
**Brand 7 (n = 2)**				
Instant	Caffeinated	Unknown	Unknown	Laboratory
Instant	Caffeinated	Unknown	Unknown	Laboratory
**Brand 8 (n = 4)**				
Cold Brew	Caffeinated	Arabica	Medium	Laboratory
Filter	Caffeinated	Arabica	Medium	Laboratory
French Press	Caffeinated	Arabica	Medium	Laboratory
Percolated	Caffeinated	Arabica	Medium	Laboratory
**Brand 9 (n = 1)**				
Boiled	Caffeinated	Blend	Medium	Home
**Brand 10 (n = 1)**				
Filter	Caffeinated	Arabica	Light	Laboratory
**Brand 11 (n = 10)**				
Cold Brew	Caffeinated	Blend	Medium	Laboratory
Filter	Caffeinated	Blend	Dark	Laboratory
Filter	Caffeinated	Blend	Light	Laboratory
Filter	Caffeinated	Blend	Medium	Laboratory
Filter	Decaffeinated	Blend	Medium	Laboratory
French Press	Caffeinated	Blend	Medium	Laboratory
Instant	Caffeinated	Blend	Medium	Laboratory
Instant	Decaffeinated	Blend	Medium	Laboratory
K-Cup	Caffeinated	Arabica	Medium	Laboratory
Percolated	Caffeinated	Blend	Medium	Laboratory
**Café or restaurant 2 (n = 2)**				
Filter	Caffeinated	Arabica	Medium	Restaurant
Filter	Decaffeinated	Arabica	Medium	Restaurant
**Brand 12 (n = 1)**				
Boiled	Caffeinated	Arabica	Dark	Home
**Brand 13 (n = 3)**				
Instant	Caffeinated	Blend	Dark	Laboratory
Instant	Caffeinated	Blend	Medium-Dark	Laboratory
Instant	Caffeinated	Unknown	Unknown	Laboratory
**Brand 14 (n = 3)**				
Instant	Caffeinated	Blend	Light-Medium	Laboratory
Instant	Caffeinated	Blend	Medium-Dark	Laboratory

Instant	Decaffeinated	Blend	Light-Medium	Laboratory
**Brand 15 (n = 3)**				
Espresso	Caffeinated	Arabica	Medium	Laboratory
Espresso	Caffeinated	Blend	Dark	Laboratory
Espresso	Caffeinated	Blend	Medium	Laboratory
**Café or restaurant 3 (n = 2)**				
Espresso	Caffeinated	Arabica	Dark	Restaurant
Espresso	Decaffeinated	Arabica	Dark	Restaurant
**Brand 16 (n = 1)**				
Filter	Caffeinated	Blend	Dark	Laboratory
**Brand 17 (n = 1)**				
Filter	Caffeinated	Arabica	Dark	Laboratory
**Café or restaurant 4 (n = 6)**				
Espresso	Caffeinated	Arabica	Dark	Restaurant
Espresso	Decaffeinated	Arabica	Dark	Restaurant
Filter	Caffeinated	Arabica	Lightn	Laboratory
Filter	Caffeinated	Arabica	Medium	Restaurant
Filter	Decaffeinated	Arabica	Medium	Restaurant
K-Cup	Decaffeinated	Arabica	Medium	Laboratory

[1] Brands 1-17: Café Najjar, Choc Full O Nuts, 8 O'Clock, Folgers, Green Mountain, Klassno, Lavazza, Loumidis, Marley, Maxwell, Mehmet, Nescafe, Nescafe Tasters' Choice, Nespresso, Peets, Safeway; Café/Restaurant 1-4: Dunkin, McDonalds, Panera, Starbucks.

Table S3. Coefficients of variation for the coffee compounds identified or tentatively identified.

Compound	Blinded QC1	Blinded QC2	Pooled QC
1-(2-Furanyl)-1,2-butanedione	5.3	3.4	8.3
N-Methyl-2-pyrrolecarboxaldehyde	13.6	8.1	16.9
3,4-Dicaffeoylquinic acid lactone		6.6	10.8
3,4-Dimethyl-1,2-cyclopentanedione	11.3	4.7	8.6
3,5-Dicaffeoylquinic acid	24.0	12.9	14.1
3-Caffeoylquinic acid	17.0	7.2	10.0
5-Caffeoylquinic acid	20.7	6.6	11.3
5-Hydroxymethylfurfural	3.3	5.9	15.2
alpha-Furyl methyl diketone isomer (i)	10.8	4.8	8.5
alpha-Furyl methyl diketone isomer (ii)	8.8	3.3	8.2
Atractyligenin	12.3	5.7	7.5
Cafamarine	17.4	11.5	13.0
Cafestol	15.8	11.3	9.0
Caffeoylquinic acid isomer (i)	14.3	7.8	10.8
Caffeoylquinic acid isomer (ii)	21.6	8.2	12.3
Caffeoylquinic acid isomer (iii)	20.5	7.9	11.7
Caffeoylquinic acid lactone isomer (i)	10.1	9.0	9.2
Caffeoylquinic acid lactone isomer (ii)	8.6	5.7	9.3
Cinnamoylglycine	6.9	3.6	7.8
Citramalic acid	13.0	6.8	9.4
Coixinden A	12.8	12.6	11.2
Coumaroylquinic acid isomer (i)	15.4	6.8	9.9
Coumaroylquinic acid isomer (ii)	18.8	21.5	14.9
Coumaroylquinic acid lactone	15.1	9.8	12.0
Cyclo(isoleucyl-prolyl)	13.5	3.6	8.0
Cyclo(isophenylalanyl-prolyl)	9.1	3.3	8.2
Cyclo(leucyl-phenylalanyl)	16.1	6.3	7.3
Cyclo(leucyl-prolyl)	9.8	3.5	10.1
Cyclo(phenylalanyl-prolyl)	9.1	3.2	5.8
Cyclo(prolyl-prolyl)	7.1	3.0	5.9
Cyclo(prolyl-valyl)	16.2	6.8	8.7
Dicaffeoylquinic acid isomer*	21.7	12.7	12.9
Feruloylquinic acid isomer (i)	21.4	6.9	10.9
Feruloylquinic acid isomer (ii)	21.2	4.8	9.6
Guanosine	14.1	7.1	10.9
Methyl-2-pyrrolecarboxaldehyde	6.9	6.6	6.1
N-p-Coumaroyltryptophan	20.7	11.4	10.7
N-Caffeoyltryptophan	18.4	9.3	12.6
Paraxanthine	10.2	9.3	11.8
Sotolone	31.4	23.9	28.4
Theobromine	10.1	4.3	8.0
Theophylline	10.6	3.6	9.4

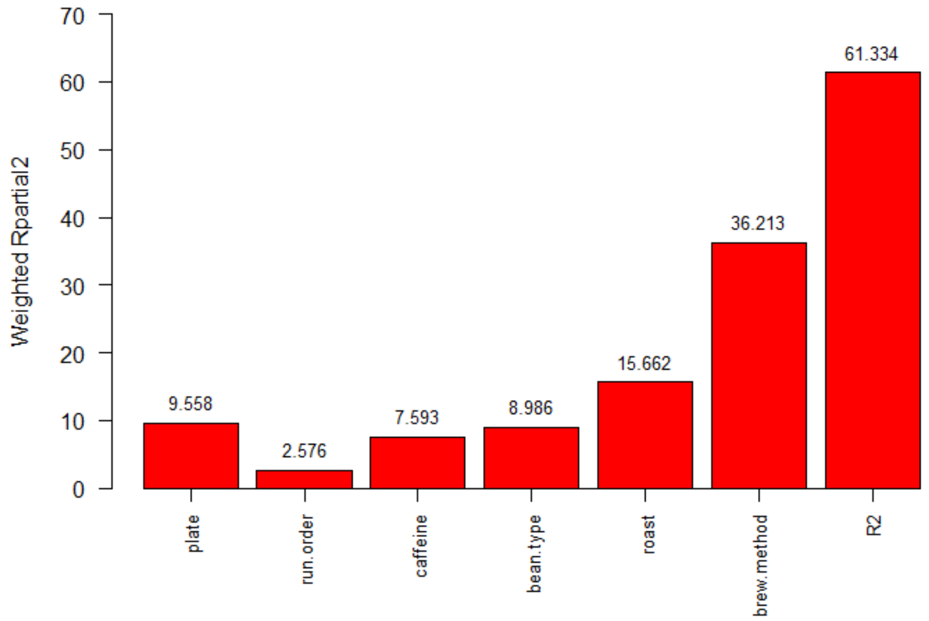

Figure S1. PC-PR2 analysis of of metabolomic data from the coffee brew samples, Principal components explaining >80% of the variability were selected.

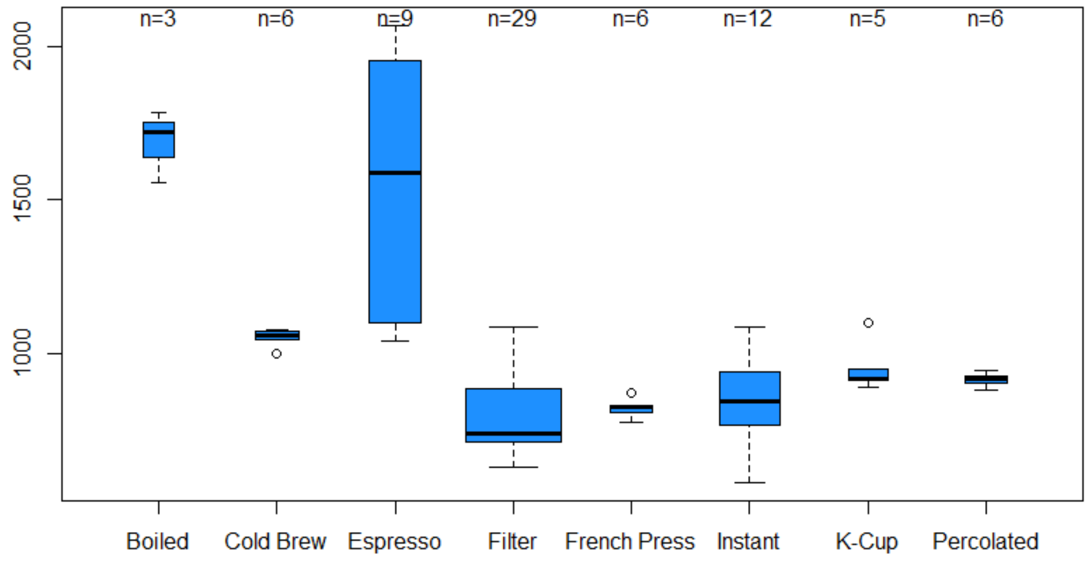

Figure S2. Boxplots showing means of sums of total intensities of mass spectrometry signals (n = 3670) for each type of coffee brews.

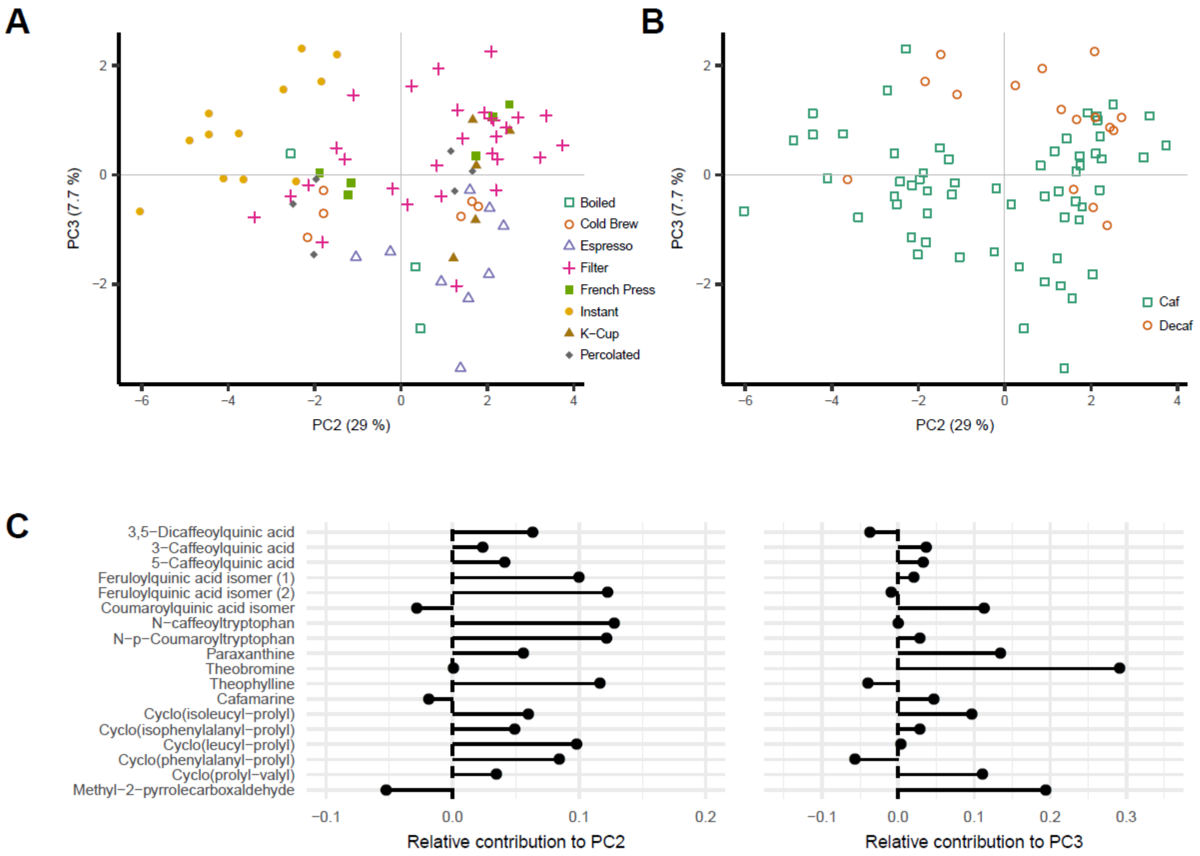

Figure S3. Principal component analysis of constituent profiles of the 18 annotated coffee compounds measured in 76 coffee brew samples. Scores on PC2 and PC3 are shown. (A) Color-coded according to type of coffee brew. (B) Color-coded according to decaffeination. (C) Loading plots.

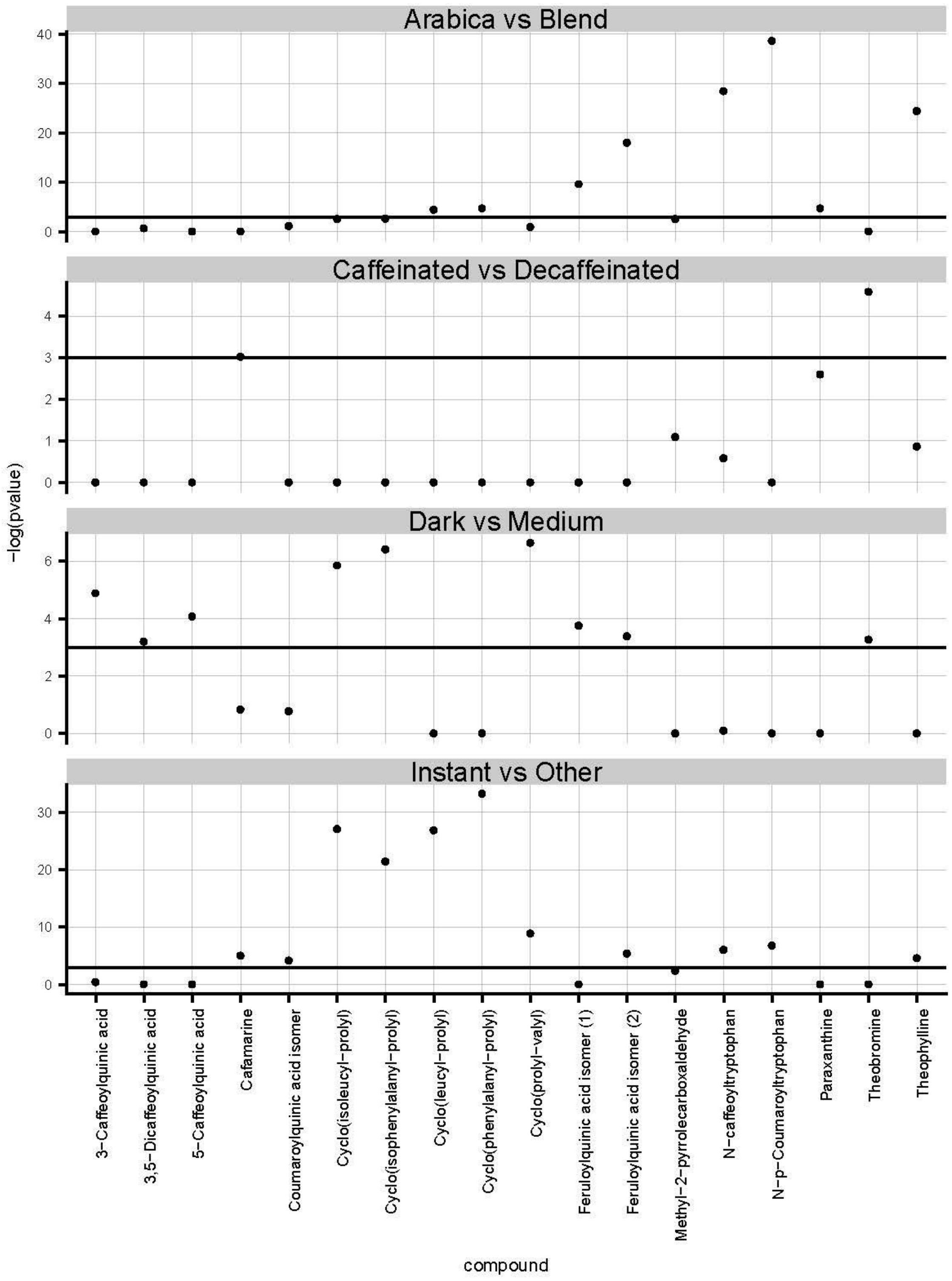

Figure S4. T-tests comparing different groups of coffee brew samples. Dark line indicates the limit for significant difference after Bonferroni correction. Comparison of dark roasted brews with medium roasted brews, decaffeinated and caffeinated coffee brew samples, coffee brews prepared with Arabica beans and blends of Arabica and Robusta beans; instant coffees to all other coffee brew samples.

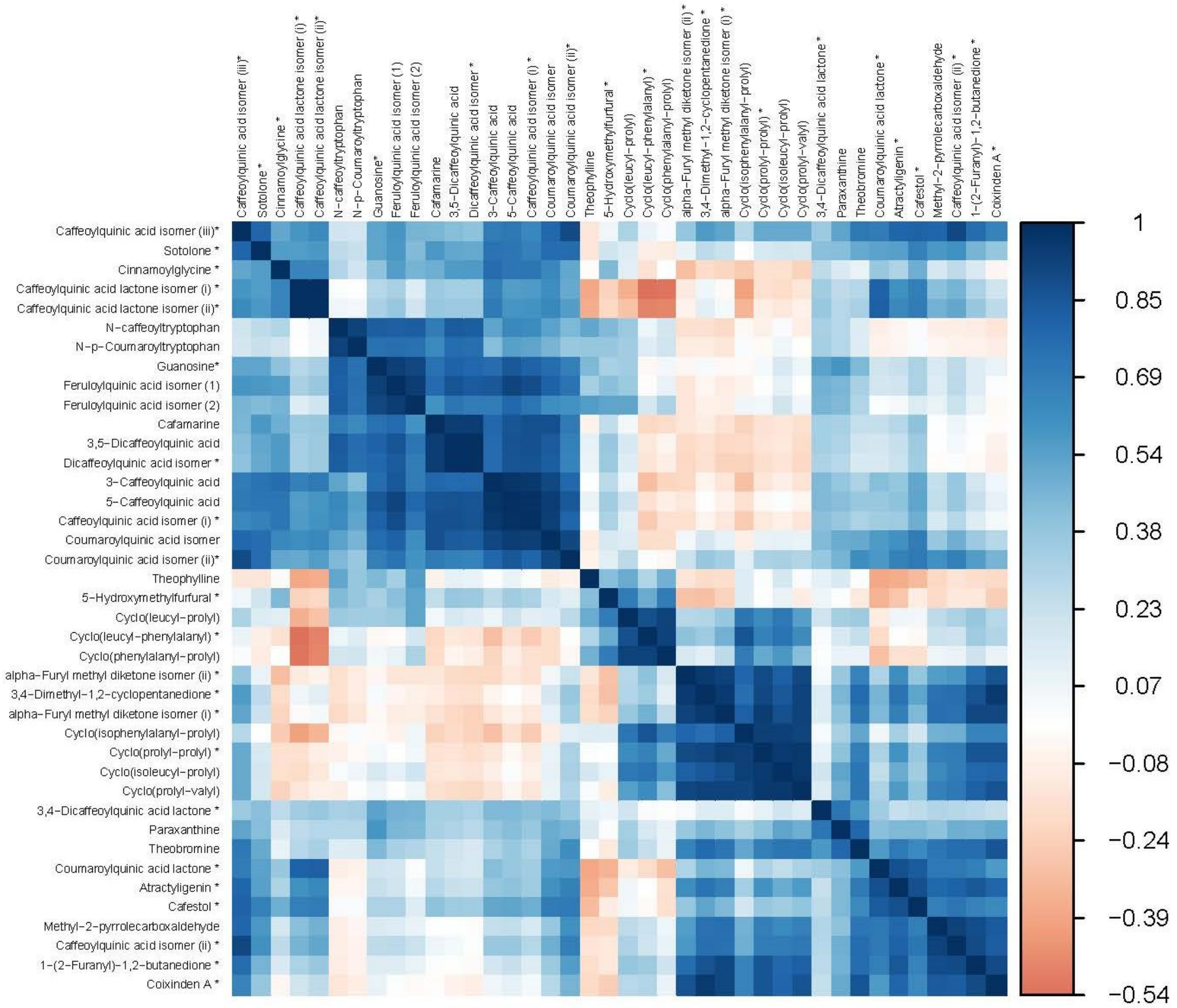

Figure S5. Heatmap showing correlations between annotated coffee compounds. See supplementary table 1 for details on annotations.
